# Providing Specialty Telehealth Care to Older, Rural Patients: Voices From Field

**DOI:** 10.1093/geroni/igab046.453

**Published:** 2021-12-17

**Authors:** Eileen Dryden, Lauren Moo

## Abstract

Older, rural adults have limited access to quality geriatric specialty care for several reasons including relatively few geriatric specialists in rural areas and lack of transportation options or patient ability to travel to more urban centers. GRECC Connect is a promising telehealth-hub and spoke model that provides rural patients access to teams of multidisciplinary geriatric specialists in more urban medical centers primarily by video connection with affiliated community-based outpatient clinics (CBOCs). This model provides a viable option for increasing access to geriatric specialty care for rural patients but is not used to the extent it could be. To date, much of our understanding of this model has come from the experts at the hub medical centers. To learn more about the experience of this model from the field we interviewed CBOC staff and providers as well as Veterans and their caregivers about geriatric specialty telehealth services. In this symposium we will discuss facilitators and barriers to implementing this model from the perspective of the field and then explore more deeply both the context of the CBOC environment and the older patient population served by rural CBOCs to further understand the challenges that are faced in attempting to connect older patients with telehealth services. Finally, we will share the perceived value of the service and alignment with local needs. This deeper understanding of the experience of the ‘spoke’ may help enhance access to much needed geriatric specialty care for rural veterans.

